# Blood Pressure Prediction Using Ensemble Rules during Isometric Sustained Weight Test

**DOI:** 10.3390/jcdd9120440

**Published:** 2022-12-07

**Authors:** Ramón Carrazana-Escalona, Adán Andreu-Heredia, María Moreno-Padilla, Gustavo A. Reyes del Paso, Miguel E. Sánchez-Hechavarría, Gustavo Muñoz-Bustos

**Affiliations:** 1 Department of Basic Science, School of Medicine, Universidad Católica de la Santísima Concepción, Concepción 4090541, Chile; 2 Departamento de Ciencias Básicas Biomédicas, Facultad de Medicina 1, Universidad de Ciencias Médicas de Santiago de Cuba, Santiago de Cuba 90100, Cuba; 3 Departamento de Psicología, Universidad de Jaén, 23009 Jaén, Spain; 4 Departamento de Ciencias Clínicas y Preclínicas, Facultad de Medicina, Universidad Católica de la Santísima Concepción, Concepción 4090541, Chile; 5 Núcleo Científico de Ciencias de la Salud, Facultad de Ciencias de la Salud, Universidad Adventista de Chile, Chillán 3780000, Chile; 6 Facultad de Ciencias de la Salud, Sede Concepción, Universidad de Las Américas, Concepción 4100000, Chile

**Keywords:** blood pressure prediction, RuleFit model, linear regression, blood pressure

## Abstract

Background: Predicting beat-to-beat blood pressure has several clinical applications. While most machine learning models focus on accuracy, it is necessary to build models that explain the relationships of hemodynamical parameters with blood pressure without sacrificing accuracy, especially during exercise. Objective: The aim of this study is to use the RuleFit model to measure the importance, interactions, and relationships among several parameters extracted from photoplethysmography (PPG) and electrocardiography (ECG) signals during a dynamic weight-bearing test (WBT) and to assess the accuracy and interpretability of the model results. Methods: RuleFit was applied to hemodynamical ECG and PPG parameters during rest and WBT in six healthy young subjects. The WBT involves holding a 500 g weight in the left hand for 2 min. Blood pressure is taken in the opposite arm before and during exercise thereof. Results: The root mean square error of the model residuals was 4.72 and 2.68 mmHg for systolic blood pressure and diastolic blood pressure, respectively, during rest and 4.59 and 4.01 mmHg, respectively, during the WBT. Furthermore, the blood pressure measurements appeared to be nonlinear, and interaction effects were observed. Moreover, blood pressure predictions based on PPG parameters showed a strong correlation with individual characteristics and responses to exercise. Conclusion: The RuleFit model is an excellent tool to study interactions among variables for predicting blood pressure. Compared to other models, the RuleFit model showed superior performance. RuleFit can be used for predicting and interpreting relationships among predictors extracted from PPG and ECG signals.

## 1. Introduction

In recent years, the arterial wave propagation theory, which relies on simultaneously collected electrocardiogram (ECG) and photoplethysmogram (PPG) signals for blood pressure (BP) estimates, has attracted the interest of many researchers [1]. This theory is based on the idea of wave propagation along a certain path. The start and end points of the path are extracted, and the pulse transit time (PTT), pulse arrival time (PAT), and pulse wave velocity (PWV) can be calculated and used to determine cardiovascular functional status (e.g., BP, arterial stiffness [2], and arterial compliance).

These similarities between the PPG wave and BP led us to speculate that it is possible to estimate BP using PPG morphological feature models, although this method requires high-quality signals because the PPG signal is sensitive to many kinds of noise. ECG–PPG features are usually used in these models because they are popular in clinical settings [3].

Several observations demonstrate that the wave amplitude (AM) and timing of wave reflections are directly related to the elastic properties of the arterial tree, as well as the stiffness index and delay between the incident and reflected wave peaks, which can be used to estimate arterial stiffness [4]. The contour of the ascending aortic pressure wave has been classified based on the reflected AM and temporal characteristics [5,6]. These classifications are in good agreement with the four age-related classes of the PPG contour [7]. Moreover, age-related trends in PPG contour triangulation have been observed [8], and the contour showed similar shape changes to the pressure wave. These results imply that the PPG contour is mainly controlled by the pressure waveform and contains cardiovascular information, such as vessel stiffness and BP. Therefore, factors that can modify the BP, such as exercise or sympathetic hyperreactivity, can affect the PPG contour as well as the relationships among hemodynamical parameters extracted from the PPG and ECG signals. Cardiovascular and sympathetic hyperreactivity partly explain the etiopathogenesis of hypertension and other cardiovascular diseases [9].

There are a number of techniques to induce sympathetic reactivity, among which the isometric stress test is one of the most widely used. It showed high predictive capability and sensitivity for diagnosing high BP [10,11]. Isometric exercise evokes a potent sympathetic reflex that triggers a significant increase in heart rate, diastolic blood pressure (DBP), and systolic blood pressure (SBP). Isometric handgrip exercise is widely used to assess autonomic disabilities [12]. However, this tool can be difficult to manipulate for persons of advanced age, those who suffer from arthritis, and those with loss of muscular function of the hand. The dynamic weight-bearing test (WBT) is a Cuban version of this tool that involves holding a 500 g weight in the left hand for 2 min. BP is taken in the opposite arm before exercise and during the last 10 s thereof [13]. This test evokes a sympathetic reflex and has been used in conjunction with heart rate variability methods to study autonomic disabilities in persons suffering from hypertension or diabetes mellitus [14].

Because the WBT induces a sympathetic response, it can be used to predict changes in BP, as well as interactions among hemodynamic parameters extracted from the PPG and ECG signals that arise during isometric exercise. There are many established regression methods, such as support vector machines (SVM), linear regression, regression trees, model trees, tree ensembles, and random forest [15]. Zadi et al. [16] designed fifth-order autoregressive moving average models to estimate BP using a PPG signal as input. Cheng et al. [17] proposed a deep learning model to predict arterial blood pressure (ABP) waveforms from PPG signals. Instead of using one PPG signal to predict BP, Fong et al. [18] used multiple PPG signals to develop an ensemble learning framework to predict BP. This model used pulse morphological parameters, time domain parameters, and PWV to achieve highly accurate results. Ensemble models have been widely used for blood glucose prediction, partly because traditional models do not always capture inter- and intra-patient contextual changes [19]. The authors of this paper propose the rule ensemble method (RuleFit), which has never previously been applied for estimating BP from PPG morphological features or PPG–ECG features. The RuleFit method is introduced in [20]. The model output is more interpretable than that of traditional ones; it is easy to ascertain the relative importance of and interactions among variables. RuleFit has been successfully applied in the fields of particle physics, medical informatics, and life sciences [21]. The aim of this study is to use the RuleFit model to measure the importance, interactions, and relationships among several parameters extracted from photoplethysmography (PPG) and electrocardiography (ECG) signals during the WBT to predict BP in healthy young subjects and to assess the accuracy and interpretability of the model output.

## 2. Material and Methods

**Figure 1:**
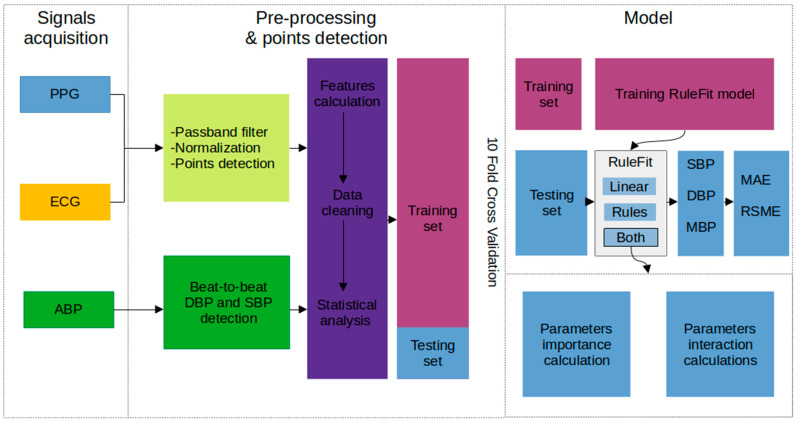
Flow chart of the experiments based on the RuleFit model. The same process was performed during rest and the weight-bearing test. Statistical analysis was done by comparing both data sets. PPG, photoplethysmography; ECG, electrocardiogram; ABP, arterial blood pressure signal; DBP, diastolic blood pressure; SBP, systolic blood pressure; MBP, mean blood pressure; MAE, mean absolute error; RMSE, root mean square error.

A flow chart of the proposed method is illustrated in Figure 1, including the data acquisition, signal pre-processing, parameter calculation, data cleaning, statistical analysis, and model training and testing steps, as well as calculation of the importance and interactions of features.

### 2.1. Data Acquisition

A non-observational crossover study of eight adolescents aged 13–18 years of age was conducted. Two subjects were excluded as they had abnormal electrocardiographic waves; thus, only six subjects were included. The participants were recruited from high schools in Jaén (Spain). They were screened for medical and developmental conditions, medication use, and learning disabilities. The inclusion criteria were as follows: (a) aged 13–18 years, (b) body mass index (BMI) > 5th percentile, and (c) no history of neurological, psychiatric, or eating disorders. This study was approved by the Ethics Committee of the Universidad de Jaén, and the procedures were in accordance with the Declaration of Helsinki (1975, as revised in 2008). Both the participants and their parents signed informed consent forms. After obtaining permission from the high school’s directors, the study was presented to each class of students, and their participation was requested. Students who were interested in taking part sent us the completed informed consent form, which was signed by the parents in the case of minors. Then, the participants were assigned to a group and a specific day on which to complete the experimental session. Weight and height were self-reported by the participants for recruitment purposes. Six high schools in Jaén participated in the study. Of all students approached, approximately 4% took part.

Sessions started at 4 p.m. They were held in a dimly lit room with controlled environmental noise and humidity and a temperature of between 24 and 27 degrees Celsius. There were no interactions or distractions between volunteers, who were allowed to rest for 15 min to adapt to the conditions. No other people were present within the experimental environment. Participants were interviewed regarding substance ingestion and physical activities before starting the experimental sessions; they were also required to be satiated (they had lunch about 1 h before) and to not have taken any caffeine.

BP was recorded continuously and non-invasively from the middle phalanx of the third finger of the left hand with a validated BP monitor (Ohmeda 2300; Ohmeda, Louisville, KY, USA) [22]. The hand was positioned at the level of the heart. ECG was obtained using an ECG100C ECG amplifier (Biopac Systems Inc., Goleta, CA, USA). Electrodes (Ag/AgCl) were placed according to Einthoven’s II derivation. A PPG100C Photoplethymogram Biopac Amplifier was used to record pulse waves. The photoplethymogram transducer (TSD200) was placed in the last phalanx of the left index finger.

Data acquisition and recording of physiological variables were carried out at 1000 Hz using a Biopac MP150 system; they were continuously recorded at rest and during the dynamic WBT. The baseline recording was performed for 2 min (at rest) in a seated position, and the dynamic WBT was also performed for 2 min while holding the 500 g weight, followed by a 2 min recovery period in the same position.

### 2.2. Signal Preprocessing, Processing, and Feature Extraction

#### 2.2.1. ECG Signals

**Figure 2:**
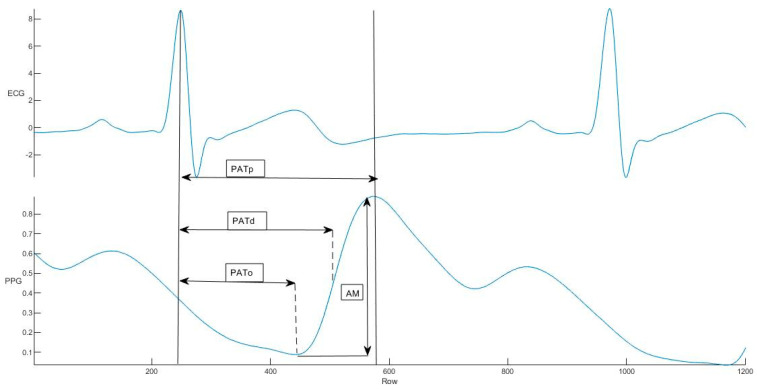
ECG and PPG signals. Pulse arrival time (PAT) for different points of the PPG signal recorded in this study.

First, each ECG signal was processed with a bandpass filter having a bandwidth of [0.5–30 Hz]. We used the Sabarimalai–Manikandan method for R-peak detection [23]. All recordings were visually examined, and R-peak misdetections were manually corrected. RR intervals were then calculated. R-peak and RR intervals were used to calculate valuable features such as PAT and relative pulse arrival time (RelPAT). Figure 2 illustrates how PAT can be calculated.

#### 2.2.2. PPG Signals

To extract meaningful information from the signals, it was necessary to normalize them [24]. The *Z-score* technique was used to normalize the signals to obtain amplitude-limited data.

(1) $$Z-score\;Normalized\;Signal=\frac{Signal-Signal\;Mean}{Standard\;Deviation\;of\;Signal}$$

Each PPG signal was processed with a bandpass filter in which the cutoff frequencies were 0.5 and 15 Hz. The purpose of this filter is to eliminate high-frequency noise and the direct current. Then, a moving average filter was applied to smooth the signal. We used the Carrazana method [25] and a fourth-order derivative algorithm for fiducial point detection on the pulse wave and its derivative and calculated a range of features from the fiducial points, i.e., the systolic peak (s), dicrotic notch (dic), and diastolic peak (dia) in the pulse wave; the point of maximum upslope on the first derivative (ms); the a, b, c, d, and e waves in the second derivative; and the early and late systolic components (p1 and p2) from the third derivative. A range of features were calculated from the fiducial points, as defined in the Supplemental Digital content (see Table, Supplemental Digital Content 1). Details of the criteria used to detect these fiducial points are provided in the table in Supplemental Digital Content 2. These features were identified from publications describing techniques for assessing arterial stiffness from pulse waves [26].

#### 2.2.3. Blood Pressure Signals

The ABP recordings were visually examined to determine whether the signal was useful for the investigation. No filtering processing was necessary because filters can modify BP values. An expert observer selected the foot and peak of the BP wave (the DBP and SBP). Mean blood pressure (MBP) was calculated by the formula $\frac{SBP+2*DBP}{3}$. BP recordings were plotted, which allowed us to identify common signal acquisition issues in the waveform [27]. These issues can be classified into two major groups, i.e., flat lines and flat peaks, which were cut out from the waveforms by simply removing part of the PPG, ECG, and BP cycle (between the start and end point of remaining flat lines) or, in the case of cycles, the full cycle.

### 2.3. Data and Statistical Analysis

#### 2.3.1. Data Cleaning

For data cleaning, the python dropna method from the pandas library was used [28]. Data cleaning is necessary to obtain meaningful results; we addressed both missing and irregular data. Each row in the dataset represents an observation of a cardiac cycle. We discarded the entire observation if it contained a single NaN value. After dealing with the missing data, we detected and corrected outliers using the *Z-score* method, which is a parametric measure with two parameters: mean and standard deviation (SD). A *Z-score* tells us how many SDs a given observation is from the mean. Outliers were detected in each column using this method. For each observation, a *Z-score* was calculated The mean and SD were calculated for each column. We discarded rows containing a *Z-score* > 3, i.e., >3 SDs from the mean. The dataset dimensions were 2970 × 58.

#### 2.3.2. Feature Selection

No feature selection process was performed. The goal of our study was not to predict BP, but rather to identify the most important variables, as well as hidden rules and interactions among variables.

#### 2.3.3. Statistical Analysis

JASP software (version 0.16) (https://jasp-stats.org, accessed on 28 January 2022) was used for the first part of the analyses, i.e., for comparing the variables before and after the WBT. All values are expressed as mean (X) and SD. All differences were considered statistically significant at *p* < 0.05. The non-parametric Wilcoxon signed-rank test (for two related samples) was used to compare variables between the rest and sustained weight conditions.

### 2.4. RuleFit

The RuleFit algorithm was used to discover hidden rules that predict beat-to-beat BP. This algorithm can transform the n-dimensional space of input features into smaller space subsets that have an explained effect on the target variable. Each rule’s influence on the predictive model and the relative importance of each independent variable can be assessed by the algorithm [29]. The RuleFit algorithm [20] fits sparse linear models that include automatically detected interaction effects in the form of binary decision rules. RuleFit fits a sparse linear model with the original features and a set of new features (decision rules). These new features capture interactions between the original features. RuleFit generates these features automatically from decision trees [30]. The RuleFit model provides a number of useful diagnostic tools, such as feature importance, partial dependence plots and feature interactions. For building the RuleFit model, RuleFit3 with R (Stanford University) was used. The tree size was 3; this permits higher order interactions. The remaining arguments were set to default values. We investigated the nature of the dependence of BP on the predictors using the tools described above.

### 2.5. Evaluation Criteria

Full (10-fold) cross-validation of the RuleFit model was performed. Cross-validation is usually the preferred method to evaluate the performance of machine learning models because it allows the model to be trained via multiple train-test splits. This provides a better indication of how well the model will perform on unseen data. To evaluate the performance of the algorithms for estimating BP, the following three criteria were applied to RuleFit models involving only linear models (no rules), only rules, and both rules and a linear model fit:

Number of terms in the model: total number of terms with a non-zero coefficient after Lasso selection. 

Mean absolute error (*MAE*): measure of the errors between paired observations expressing the same phenomenon; the average absolute difference between the prediction and actual observation is calculated over the test sample, where all individual differences have equal weight.

(2) $$MAE=\frac{1}{N}\overset{N}{\underset{1}{\sum }}(X_{p}-\bar{X_{p}})$$

where *N* is the sample size, *Xp* is the predicted value, and bar*Xp* is the true value.

Root mean squared error (*RSME*): the *RMSE* is the *SD* of the residuals (prediction error). Residuals are a measure of how far away the data points are from the regression line; *RMSE* is a measure of the dispersion of the residuals.

(3) $$RSME=\sqrt{\frac{\sum_{1}^{N}{\left(X_{p}-\bar{X_{p}}\right)}^{2}}{N}}$$

where *N* is the sample size, *Xp* is the predicted value, and bar*Xp* is the true value.

### 2.6. RuleFit Model Interpretation

#### 2.6.1. Importance

To quantify the relative contribution of every base learner to the predictions of the final ensemble, importance can be calculated. Friedman and Popescu [20] defined the importance of a linear term as:

(4) $$I_{j}=b_{j}\vee \cdot sd\left(l_{j}\left(x_{j}\right)\right)$$

where ‘*sd*’ denotes the sample standard deviation. Similarly, the global importance of a rule is given by: $I_{k}=a_{k}\vee \sqrt{s_{k}\left(1-s_{k}\right)}$, where $\sqrt{s_{k}\left(1-s_{k}\right)}$ is the sample standard deviation of rule ‘*k*’, the support of rule ‘*k*’ in the training data, or the proportion of training observations to which rule ‘*k*’ applies:

(5) $$s_{k}=\frac{1}{N}\overset{N}{\underset{i=1}{\sum }}r_{k}\left(x_{i}\right)$$

#### 2.6.2. Interactions

Prediction rules are well-suited for capturing interaction effects of input variables. However, non-zero coefficients for rules involving multiple predictor variables in the final ensemble are a necessary but not sufficient condition for determining the presence of interaction effects. For example, the interaction may be cancelled out by other rules in the ensemble, or a rule involving multiple predictor variables may reflect only the main effects of (correlated) predictor variables and not interaction(s). Friedman and Popescu [20] developed a statistic for assessing whether a predictor variable is involved in interactions with other predictor variables in the model. The underlying rationale is that, in the presence of interaction effects, the effects of individual predictor variables are not additive.

## 3. Results

The overall performance of the proposed and comparison methods was validated over 1721 beats (Rest: 1026, WBT: 695) from six subjects who were in supine and seated resting positions. Table 1 shows that, among the hemodynamic parameters studied, significant differences were only found in the mean BP, SBP, dicrotic PAT, PWV, onset–onset wave interval, and peak–peak wave interval. We included all variables in this analysis (see Table, Supplemental Digital Content 4).

### 3.1. Blood Pressure Appeared to Be Highly Nonlinear with Some Interaction Effects

Table 2 shows the RuleFit model evaluation criteria. Applying RuleFit to these data (DBP) produced a model involving 303 (rules + linear) terms with non-zero coefficients. The average MAE was 1.95, as estimated by 10-fold cross validation. The corresponding error for an additive model restricted to main effects only (L = 2) was 2.056 and that for a model involving only linear terms was 2.303. Thus, including additive nonlinear terms to the model improved prediction accuracy by approx. 11% relative to a purely linear model, while including interaction led to a further improvement of approx. 5%. Thus, the target function appears to be highly nonlinear, with some interaction effects. The same analysis was applied to other response variables in both states. It can be seen that the RuleFit model improved prediction accuracy relative to a linear model and a model including interaction effects. The prediction accuracy was higher for DBP than SBP and MBP in both states, although there were more terms with non-zero coefficients for DBP.

### 3.2. Feature Importance and Interactions

**Figure 3:**
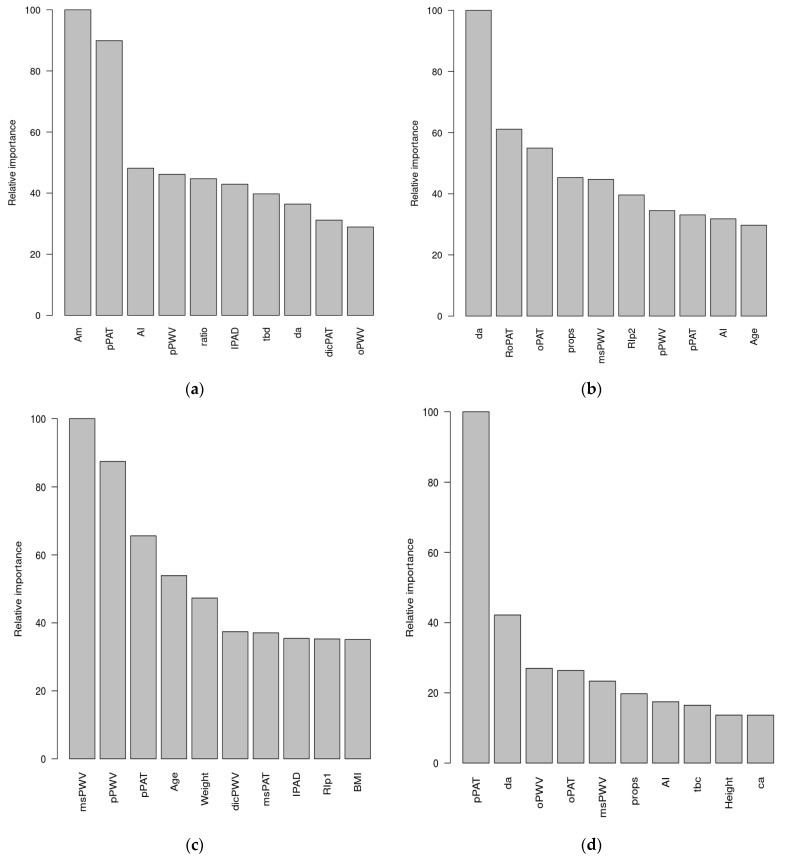
Relative importance of the 10 most important input predictor variables for (**a**) DBP during rest, (**b**) DBP during the WBT, (**c**) SBP during rest, and (**d**) SBP during the WBT.

Figure 3 shows the relative importance of the ten most important input predictor variables as averaged over all predictions in descending order of estimated importance. If the DBP is analyzed in a resting state, it is observed that the AM is the most important variable followed by the peak pulse arrival time (pPAT). The SBP shows that, along with demographic variables such as age, weight, and BMI, the parameters derived from PAT and PWV are the most important.

Ten globally important terms resulting from the RuleFit model were analyzed with respect to their estimated importance (see Table, Supplemental Digital Content 3) to demonstrate that the rules are more important than linear terms. The complex relationships among variables can explain BP variations.

PAT and PTT are often used to predict BP. Our data suggest that PAT has the strongest effect on BP. However, PWV had a stronger effect on SBP. This might be because PWV is associated with rapid changes in the cardiovascular system, as well as SBP. However, both DBP and PAT are associated with slow changes; this explains why PAT seems to have a stronger effect on DBP, and PWV on SBP, i.e., because their changes are more synchronized with each other.

We also analyzed MBP; AM was the most important variable, followed by the PAT. However, during the WBT, the most important variables were pPAT, RoPAT, DA, and slope BC. Analysis of the distribution of extreme BP values (10% lower or higher values) was carried out to determine the relative importance of the predictors. The results of the analysis of the distribution of extreme DBP, SBP, and mean BP values, at rest (see Figure, Supplemental Digital Content 5–7) and during the WBT (see Figure, Supplemental Digital Content 8–10), are provided in the Supplemental Digital Content.

**Figure 4:**
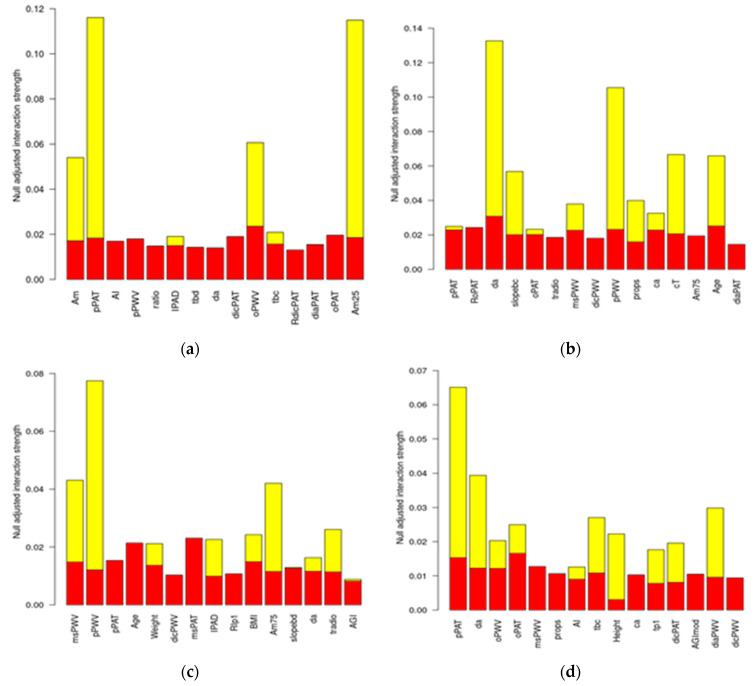
Interactions among variables. (**a**) DBP during rest; (**b**) DBP during the WBT; (**c**) SBP during rest; (**d**) SBP during the WBT.

The presence of interaction effects is always important because it shows the researchers how two or more variables work together to impact the dependent variable and can explain more of the variance therein. Figure 4 shows the values of H, along with the corresponding null SD, for the most important variables in the dataset. Most of these variables appear to interact with other variables, although the size of the effects is not large. In the resting state, with DBP as the target variable, pPAT and AM25 showed some interactions (albeit weak) with other variables. The WBT induced interactions among variables, with DA and pPWV showing the strongest interactions with the other variables (with DBP as the target variable). The interactions among variables with MBP as the target can be found in the Supplemental Digital Content (see Figure, Supplemental Digital Content 11). In summary, although weak (<10%), there were interactions between variables that RuleFit captures, which explained some of the variance in the dependent variables.

## 4. Discussion

Linear models are easy to quantify and describe, but linear regression models do not account for interactions between features. RuleFit addresses this issue; it is as simple and interpretable as linear models but integrates interactions [29]. However, no studies have previously examined the applicability of RuleFit to interactions of ECG and PPG features or its utility for predicting beat-to-beat BP.

As shown in Table 2, model improvement was achieved by including both interaction and rules terms. This suggests that BP is highly nonlinear and influenced by interaction effects. This study showed that the relationship between BP and hemodynamic parameters cannot be fully explained by linear models. This is in good agreement with other studies done in this field, such as [3,31], which found nonlinear relationships of BP and PWV with PAT features. Furthermore, the PPG-based BP estimation technique has not been widely accepted yet for BP monitoring because it involves parameters closely related to personal arterial characteristics [3,32]. A more detailed analysis revealed interactions among the features, although these were weaker. A lack of literature studies analyzing interactions between BP and PPG features makes comparisons impossible. However, studies comparing linear and more complex models are available and report complex relationships between these features [31,32].

A comparison between our work and the literature is shown in Table 3. MAE was the main evaluation metric in most studies. The MAE is a linear measure in which all individual differences are weighted equally in the average. The MAE values obtained in this study were smaller than those in [27,33,34], indicating that RuleFit could serve as a powerful tool to predict beat-to-beat BP. However, a comparative study of the previous models and our model is needed (using the same dataset to avoid any influence of data variability).

Another important result in this study is that, during the WBT, the RuleFit model showed poorer performance during the resting state. This may be linked to changes in sympathetic activity. In fact, changes in vascular properties regulated by sympathetic activity, such as vessel radius and vasomotor tone, can be pronounced during exercise. Even if RuleFit takes into account feature interactions, as a generalized linear model it is susceptible to changes in linear relations between target and independent variables. For instance, the relationship between PAT and MBP is treated as linear for a short period of time when vascular properties are assumed to be stable, so the model must be adjusted frequently to accommodate changes in vascular properties [35,36]. Hence, during the WBT, variable relationships may become too complex for explication by linear models. However, as can be seen in Table 2, changes in performance were not dramatic, showing the potential of RuleFit to fit a model including variables that can explain more of the variance in the response variable.

### Limitations

There were some limitations to this study. First, BP measurements using finger cuffs are less accurate than those using invasive arterial lines or brachial cuffs. However, measurement of BP using these methods presents some difficulties; although accurate, brachial cuffs cannot instantaneously and continuously measure BP, and arterial lines are invasive and thus ethically less acceptable for healthy subjects. A second WBT requires that the subject hold a weight for some time, which is uncomfortable and may lead to motion artifacts. Although this was corrected by removing signal segments with motion artifacts, valuable information was lost. Third, the sample size (N = 6) in this study was small, making the model vulnerable to overfitting. This might explain the better performance of the RuleFit evaluation criteria compared to other models; however, SD was not used as a measure of error in most of the papers analyzed, and comparison cannot be done using the mean alone. The main aim of this study, however, was not to achieve precise BP predictions, but rather to explore the utility of RuleFit for BP prediction, and the contributions of and interaction among features. Fourth, we did not use feature selection because we wanted to determine the contribution of all features to the model. Future work could investigate whether feature selection improves model performance on a subject-to-subject basis, where this study showed that feature contributions to the model are highly variable among subjects.

## 5. Conclusions

In conclusion, the present study demonstrated the potential of the RuleFit model for predicting beat-to-beat BP using PPG and ECG signals from healthy subjects during rest and the WBT. During the WBT, BP values increased, which was followed by statistically significant changes in some of the hemodynamical parameters extracted from PPG and ECG. However, the majority of the hemodynamical parameters did not change. The present study also found that (1) BP appeared to be highly nonlinear, with some interaction effects; (2) the RuleFit model improved prediction accuracy over a linear model; (3) rule terms were the most important terms; (4) derivatives of PAT and PWV, as well as the AM of the pulse wave, play a fundamental role; and (5) BP prediction from PPG parameters is strongly linked to individual differences in the response to exercise. The RuleFit model is an excellent tool to study interactions among variables to predict BP. Compared to the other models studied, RuleFit showed good performance and can be used for predicting and interpreting relationships among predictors extracted from PPG and ECG signals. Moreover, RuleFit revealed the interaction changes among hemodynamical parameters during the WBT compared to rest during BP prediction.

Future research: In this work, we used RuleFit to reveal hidden rules and interactions among hemodynamical parameters extracted from ECG and PPG. A notable result of the present study was that the relative importance of parameters (and their interactions) for BP prediction was highly variable among subjects. In further work on BP prediction, we will analyze the interactions among these parameters for all subjects individually. Moreover, a larger sample will be recruited, and the same analysis will be applied to patients with cardiovascular diseases, such as hypertension. Furthermore, we will conduct a comparative study of the prediction of BP using our dataset involving several models mentioned in the literature.

## Figures and Tables

**Table 1:** Effect of the dynamic weight-bearing test on hemodynamic parameters. DBP, diastolic blood pressure; SBP, systolic blood pressure; MBP, mean blood pressure; AM, amplitude; Tonset, pulse arrival time onset; Tpeak, pulse arrival time peak; Tdiff, pulse arrival time maximal slope; TonsRel, relative pulse arrival time onset; TpeRel, relative pulse arrival time peak; TdiRel, relative pulse arrival time maximal slope; PWVons, pulse wave velocity from Tonset; PWVpeak, pulse wave velocity from Tpeak; PWVdiff; pulse wave velocity from maximal slope; cT, crest time; X, mean; SD standard deviation. * Statistically significant difference at *p* < 0.05.

	Rest		WBT		
Variables	X	SD	X	SD	*p*
DBP (mmHg)	76.47	3.23	82.69	4.58	0.06
SBP (mmHg)	126.79	9.74	138.02	14.33	0.03 *
MBP (mmHg)	93.25	27.1	101.13	5.41	0.03 *
Am	2.79	0.24	2.9	0.2	0.09
oPAT (ms)	0.25	0.06	0.23	0.05	0.09
pPAT (ms)	0.28	0.04	0.29	0.04	1
diaPAT (ms)	0.55	0.02	0.54	0.02	0.03 *
RoPAT	0.36	0.08	0.34	0.05	0.56
RpPAT	0.4	0.08	0.43	0.07	0.03 *
oPWV (s/m)	6.42	1.35	6.88	1.04	0.09
pPWV (s/m)	5.6	0.87	5.37	0.83	1
DiaPWV (s/m)	2.83	0.1	2.88	0.08	0.03 *
dO (ms)	0.71	0.07	0.68	0.06	0.03 *
Dp (ms)	0.72	0.07	0.69	0.06	0.03 *
cT (ms)	0.09	0.01	0.09	0.01	0.03 *

**Table 2:** Evaluation criteria for the comparison models. MAE, mean absolute error; RMSE, root mean square error; WBT, weight-bearing test; DBP, diastolic blood pressure; SBP, systolic blood pressure; MBP, mean blood pressure. Rules refers to the number of rules obtained from the model.

Model	State	Stats	DBP (mmHg)	SBP (mmHg)	MBP (mmHg)
Linear + Rules	Rest	Rules	303	194	208
		MAE	1.95	3.38	2.18
		RSME	2.69	4.72	2.87
	WBT	Rules	304	355	194
		MAE	2.67	3.51	2.695
		RSME	4.01	4.59	3.79
Linear	Rest	Rules	48	57	56
		MAE	2.30	3.60	2.39
		RSME	3.19	4.86	3.16
	WBT	Rules	36	54	47
		MAE	3.32	4.9	3.40
		RSME	4.57	5.39	4.50
Additive model (main effects only, i.e., only rules)	Rest	Rules	655	338	612
		MAE	2.06	3.47	2.29
		RSME	2.89	4.79	3.02
	WBT	Rules	581	569	563
		MAE	2.87	3.76	2.98
		RSME	4.09	4.92	4.16

**Table 3:** Comparison of the results of this work with related works. SVM, support vector machine; SBP, systolic blood pressure; DBP, diastolic blood pressure; MBP, mean blood pressure; MAE, mean absolute error; RSME, root mean square error; WBT, weight-bearing test.

Author	Method Used	Evaluation Metrics	SBP (mmHg)	DBP (mmHg)	MBP (mmHg)
[33]	Neural network	MAE	12.38	6.34	
[35]	Multiple nonlinear regression	MAE	5.67		
[27]	Deep learning	MAE	9.43	6.88	
[34]	SVM	MAE	11.89	8.83	
[36]	Deep learning (LSTM)	RSME	3.73	2.43	
This work	RuleFit (Rest)	MAE RSME	3.38 4.72	1.95 2.68	2.18 2.87
	WBT	MAE RSME	3.55 4.59	2.67 4.01	2.69 3.79

## Data Availability

Not applicable.

## Supplementary Materials

The following supporting information can be downloaded at: https://www.mdpi.com/article/10.3390/jcdd9120440/s1, Supplemental Digital Content 1: Feature extraction from PPG and ECG signals; Supplemental digital content 2: The criteria listed in the table below; Supplemental Digital Content 3 Ten globally most important terms resulting from the RuleFit model in order of their estimated importance; Supplemental Digital Content 4: Effect of the Dynamic weight-bearing test on the hemodynamic parameters; Supplemental digital content 5: DBP importance predictors during rest. (a) All values; (b) one subject values; (c) Lowest 10% values; (d) Highest 10% values were used to identify fiducial points on PPG pulse waves; Supplemental digital content 6: SBP importance predictors during rest. (a) All values; (b) one subject values; (c) Lowest 10% values; (d) Highest 10% values; Supplemental digital content 7: MBP importance predictors during rest. (a) All values; (b) one subject values; (c) Lowest 10% values; (d) Highest 10% values; Supplemental digital content 8: DBP importance predictors during WBT. (a) All values; (b) one subject values; (c) Lowest 10% values; (d) Highest 10% values; Supplemental digital content 9: SBP importance predictors during WBT. (a) All values; (b) one subject values; (c) Lowest 10% values; (d) Highest 10% values; Supplemental digital content 10: MBP importance predictors during WBT. (a) All values; (b) one subject values; (c) Lowest 10% values; (d) Highest 10% values; Supplemental digital content 11: Interaction among features for MBP (a) in rest state; (b) during WBT
