# Supplementary material for: Blood Pressure Prediction Using Ensemble Rules during Isometric Sustained Weight Test
Source: J Cardiovasc Dev Dis. 2022 Dec 7;9(12):440. doi: 10.3390/jcdd9120440 (PMC9781478; doi:10.3390/jcdd9120440)

Supplemental Digital Content

Supplemental Digital Content 1. Feature extraction from PPG and ECG signals

Extraction aspect	Feature notation	Description	Number
	dO	Time from 2 consecutive foot wave	1
	dP	Time from 2 consecutive peak wave	2
	dT	t(dia) - t(s)	3
	cT	t(s)	4
	props	t(s)/T	5
Waveform,x Timings	tsys	t(dic)	6
	tdia	T - t(dic)	7
	tratio	t(s)/t(dic)	8
	propdt	(t(dia) - t(s))/T	9
	tp1-dia	t(dia) - t(p1)	10
	tp2-dia	t(dia) - t(p2)	11
	IPR	60/T	12
	Am25	Time from onset to the 25% of Amplitud	13
	Am50	Time from onset to the 50% of Amplitud	14
Amplitudes	Am75	Time from onset to the 75% of Amplitud	15
	Am	Amplitud of Pulse wave	16
	AI	(x(p2) - x(p1))/x(s)	17
	RI	x(dia)/x(s)	18
Areas	RIp1	x(dia)/x(p1)	19
	RIp2	x(dia)/x(p2)	20
	ratio p2-p1	x(p2)/x(p1)	21
	A1	area from pulse foot to dicrotic notch	22
	A2	area from dicrotic notch to pulse end	23
	IPA	A2/A1	24

First derivative(x') Amplitudes	ms	x'(ms)/x(s)	25
PPG'',x'' Amplitudes	b/a	x''(b)/x''(a)	26
	c/a	x''(c)/x''(a)	27
	d/a	x''(d)/x''(a)	28
	e/a	x''(e)/x''(a)	29
	AGI	(x''(b) - x''(c) - x''(d) - x''(e))/x''(a)	30
	AGIint	(x''(b) - x''(e))/x''(a)	31
	AGImod	(x''(b) - x''(c) - x''(d))/x''(a)	32
Timings	tbc	t(c) - t(b)	33
	tbd	t(d) - t(b)	34
Slopes	slopebc	d/dt of straight line between b and c, normalized by a	35
	slopebd	d/dt of straight line between b and d, normalized by a	36
Combined	Multiple	IPAD	37
ECG-PPG	PAT	oPAT	38
		pPAT	39
		msPAT	40
		dicPAT	41
	PWV	diaPAT	42
		oPWV	43
		pPWV	44
	Relative PAT (PAT/RR)	msPWV	45
		dicPWV	46
		diaPWV	47

	RR	RoPAT<br>RpPAT<br>RmsPAT<br>RdicPAT<br>RdiaPAT<br>RR interval	48<br>49<br>50<br>51<br>52<br>53
Demographic features		Age<br>Sex<br>Height<br>Weigh<br>IMC	54<br>55<br>56<br>57<br>58

Supplemental digital content 2

The criteria listed in the table below were used to identify fiducial points on PPG pulse waves.

Signal	Fiducial point	Criterion
PPG,x	0	0
	0	0
	0	0
PPG’,x’	0	0
PPG”, x”	0	0
	0	0
	0	The greatest maximum of x” between b and e (or if no maxima then the first of (i) the first maximum on x’ after e, and (ii) the first minimum of x’” after e).
	0	0
	0	The second maximum of x” after ms and before 0.6 T (unless the c wave is an inflection point, in which case take the first maximum).
	0	0
PPG’”, x’”	0	0
	0	Identify a candidate p2 at the last local minimum of x’” before d

	(unless c = d, in which case take the first local minimum of x''' after d). If there is a local maximum of x between this candidate<br>and dic then use this instead.

Supplemental Digital Content 3 Ten globally most important terms resulting from the RuleFit model in order of their estimated importance

State	DBP			
	Imp.	Coeff.	Sup.	Rule
Rest	100	-2.270	0.101	0.224<pPAT<0.246 & cT>0.091
	75.26	-1.128	0.297	pPAT >0.247 & AI < -0.451
	70.14	-1.699	0.087	Height > 1.575 & 0.222<pPAT <0.246 & 1.92<Am <3.68
	66.24	0.007		A2
	59.42	20.66		tbd
	55.28	2.69		RIp1
	54.09	-2.99	0.015	Am25>0.370 & Am50<0.129
	51.33	0.742	0.339	Da<-0.149
	47.53	-0.664	0.401	BMI >19.30 & Am >2.64
	46.07	0.728	0.250	AGI < -1.14 & RoPAT >0.349
WBT	100	-2.25	0.57	Weight>45.95 & dicPAT <0.522
	98.02	-2.30	0.66	0.202<oPAT<0.317
	89.89	2.08	0.64	Age<16 & A1 >121,2 & RoPAT<0.43
	87.89	4.54		RIp2
	85.67	2.06	0.30	5.899<msPWV<6.373 & AI >-0.538
	75.34	3.48	0.06	Da < 0.124 & RpPAT<0.336

	73.73	-1.64	0.48	Age> 14.50 & Am25>0.354
	73.20	-1.62	0.49	Age>14.50 & RoPAT < 0.404
	72.52	2.10	0.17	A1>144. 6 & da < -0.055
	67.62	1.94	0.18	MsPWV >6.322 & slopebc > 0.018
	SBP			
Rest	100	-2.056	0.331	BMI<19.30 & Am75 <0.242
	87.82	1.705	0.540	Weight >42.6 & oPWV >4.832
	83.54	-1.788	0.286	Age <14.5 & Am75 <0.225
	73.09	-1.65	0.240	PPWV<4.992 & AGI < -0.883
	67.83	-1.31	0.464	MsPWV <6.2
	65.76	1.323	0.363	MsPAT < 0.258 & tradio >0.301
	53.70	-1.737	0.099	DiaPWV <2.717 & RdicPAT <0.634
	53.21	1.135	0.289	Age > 16 & oPAT <0.322
	49.87	-1.901	0.069	dP>0.772 & 0.0041 < slopebd < 0.0046
	49.34	1.075	0.270	42.6<Weight <61 & Am >2.005
WBT	100	2.34	0.610	Weight > 42.60 & props > 0.1047 & AGIint <1.53
	91.21	-2.41	0.248	OPAT>0.220 & pPAT>0.292
	82.24	-28.87		Height
	77.22	2.80	0.112	PPAT<0.278 & tbc < 0.092
	74.22	1.707	0.456	0.226<pPAT<0.309 & diaPAT <0.551

	72.82	-1.723	0.375	oPAT >0.216 & pPAT >0.230
	53.84	-1.32	0.322	Age>16 & ca <0.201
	51.48	-1.28	0.361	OPAT>0.215 & da>-0.181
	47.99	-1.32	0.221	AGE>16 & da >-0.111
	46.96	-1.50	0.850	Ca <0.334
	MBP			
Rest	100	0.021	0.049	0.301<oPAT<0.32
	86.03	-0.008	0.623	OPAT>0.208 & pPAT >0.222 & Am>1.763
	85.41	0.020	0.040	MsPAT<0.256 & pPWV >5.675
	76.80	0.0153	0.057	PPAT<0.235 & cT <0.091
	74.16	-0.010	0.128	MsPWV<6.37 & tbc >0.885 & AGIint > -1.21
	73.19	0.008	0.777	pPAT<0.341 & diaPAT<0.579
	72.95	0.015	0.051	Age >1.76 & ca > 0.197
	67.65	-0.009	0.132	0.224<PPAT < 0.274 & cT>0.090
	67.20	-0.018	0.028	Am25>0.451 & AGImod < -1.15
	66.10	0.0005		A2
WBT	100	-2.56	0.29	OPAT>0.220 & RoPAT<0.424
	97.27	3.95	0.09	PPAT<0.235 & slopebc >0.011
	66.78	-2.17	0.15	MsPWV <5.85 & RoPAT<0.448
	62.20	-0.484		Age

	45.56	-1.19	0.739	Tradio >0.265 & da>-0.186
	43.17	1.319	0.177	DiaPAT < 0.548 & pPWV >5.324
	43.06	-1.131	0.177	CT<0.075
	42.88	1.061	0.667	pPWV<7.007 & da >-0.168
	41.61	1.633	0.097	Tradio <0.273 & slopebc >0.018
	39.38	-2.335	0.040	Tradio <0.2737 slopebc <0.017

.

Supplemental Digital Content 4. Effect of the Dynamic weight-bearing test on the hemodynamic parameters, * Statistically significant difference at $p < 0.05$.

	Rest		WBT		
Feature	X	SD	X	SD	p
oPAT	0.25	0.06	0.23	0.04	0.09
pPAT	0.28	0.04	0.29	0.03	1
msPAT	0.26	0.02	0.25	0.01	0.09
dicPAT	0.5	0.02	0.5	0.02	0.22
diaPAT	0.55	0.02	0.54	0.02	0.03 *
oPWV	6.42	1.23	6.88	0.95	0.09
pPWV	5.6	0.79	5.37	0.75	1
msPWV	6.09	0.33	6.2	0.32	0.16
dicPWV	3.09	0.09	3.14	0.13	0.22
diaPWV	2.83	0.09	2.88	0.07	0.03 *
dO	0.72	0.06	069	0.06	0.03 *
dP	0.72	0.06	0.69	0.06	0.03 *
dT	0.24	0.03	0.23	0.02	0.56
cT	94.29	11.46	0.09	0.01	0.03 *
tsys	0.28	0.01	0.28	0.01	0.31
props	0.13	0.02	0.14	0.02	0.44
tdia	0.43	0.05	0.41	0.05	0.06

tradio	0.34	0.05	0.34	0.04	0.84
propdT	0.33	0.03	0.34	0.04	0.56
tp1	0.22	0.02	0.22	0.01	0.56
tp2	0.13	0.01	0.13	0.01	1
IPR	0.08	0.01	0.09	0.01	0.06
Am100	2.79	0.22	2.9	0.19	0.09
Am25	0.43	0.08	0.42	0.06	0.56
Am50	0.29	0.09	0.28	0.09	0.44
Am75	0.15	0.05	0.15	0.05	0.69
AI	-0.37	0.16	-0.37	0.13	0.69
RI	0.19	0.09	0.16	0.11	0.22
RIp2	0.3	0.09	0.24	0.15	0.22
RIp1	0.2	0.1	0.16	0.11	0.22
ratio	0.6	0.19	0.6	0.16	0.84
A1	0.24	0.03	0.24	0.03	0.44
A2	0.24	0.03	0.23	0.02	1
IPA	1.03	0.03	1.1	0.14	0.44
tbc	0.09	0.01	0.09	0	0.31
tbd	0.15	0.01	0.14	0.02	0.06
slopebc	0.01	0	0.01	0	0.69
slopebd	0.01	0	0.01	0	0.31
ba	-1.07	0.12	-1.07	0.15	1
ca	0.13	0.08	0.13	0.11	0.84
da	-0,12	0.05	-0.13	0.04	0.56
ea	0.19	0.06	0.19	0.06	0.69
AGI	-1.28	0.22	-1.26	0.26	0.69
AGIint	-1.26	0.17	-1.26	0.21	1
AGImod	-1.09	0.18	-1.07	0.21	0.56
ms	0	0	0	0	0.69
IPAD	-1.13	0.05	-1.13	0.06	0.84
DBP	76.47	3.23	82.69	4.58	0.06
SBP	126.79	9.74	138.02	14.33	0.03 *

MBP	93.25	27.1	101.13	5.41	0.03 *
RR	0.72	0.06	0.69	0.06	0.03 *
RoPAT	0.36	0.07	0.34	0.05	0.56
RpPAT	0.4	0.07	0.43	0.07	0.03 *
RmsPAT	0.36	0.03	0.37	0.03	0.22
RdicPAT	0.71	0.04	0.73	0.04	0.16
RdiaPAT	0.78	0.05	0.79	0.06	0.09
HR	84.84	7.18	88.26	6.77	0.06

Supplemental digital content 5. DBP importance predictors during rest. (a) All values; (b) one subject values; (c) Lowest 10% values; (d) Highest 10% values

(a)

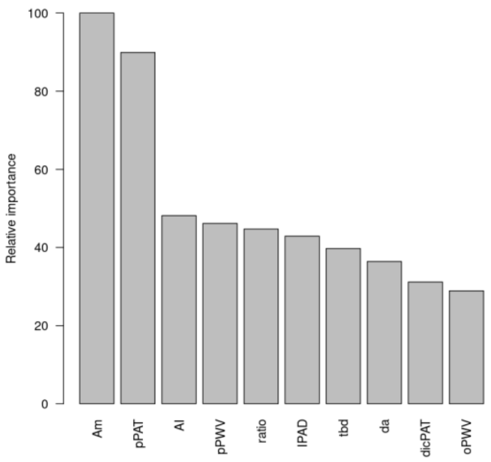

(b)

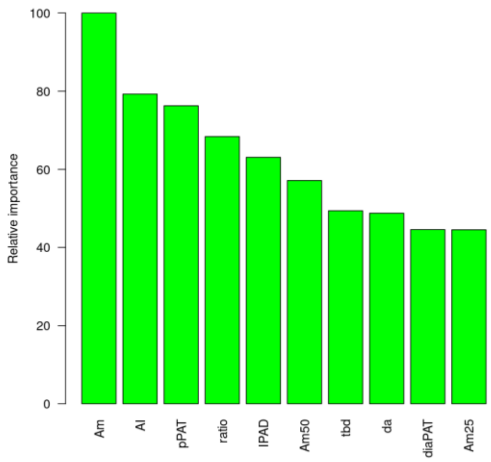

(c)

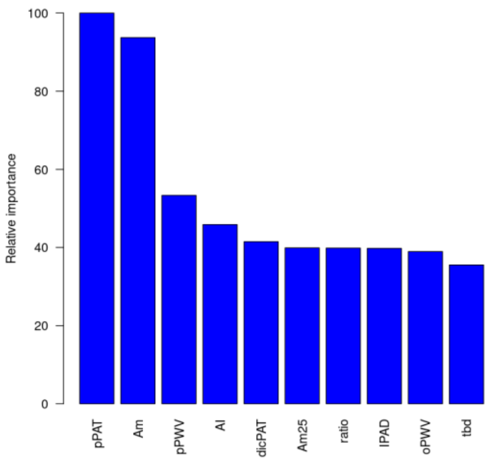

(d)

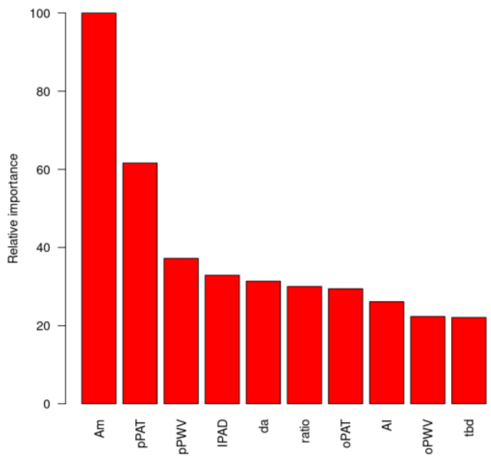

Supplemental digital content 6. SBP importance predictors during rest. (a) All values; (b) one subject values; (c) Lowest 10% values; (d) Highest 10% values

(a)

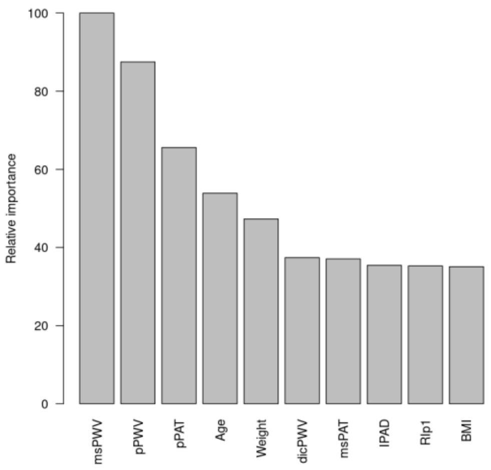

(b)

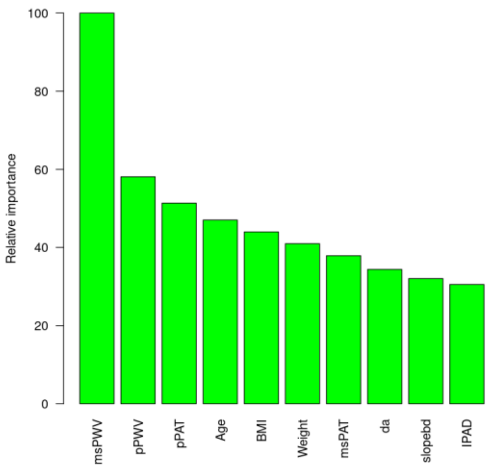

(c)

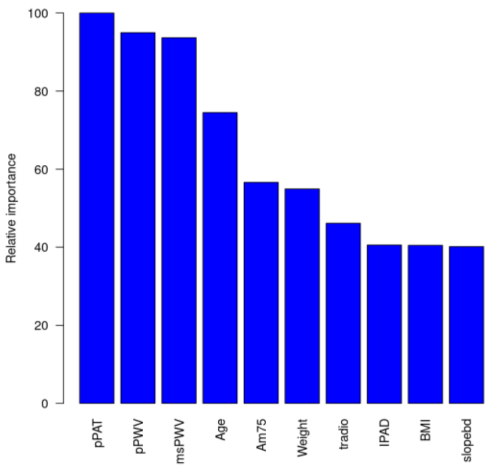

(d)

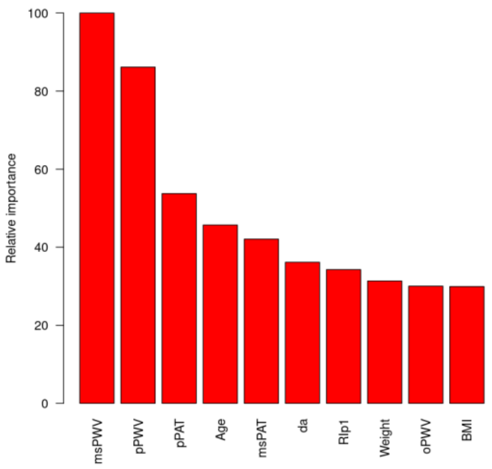

Supplemental digital content 7. MBP importance predictors during rest. (a) All values; (b) one subject values; (c) Lowest 10% values; (d) Highest 10% values

(a)

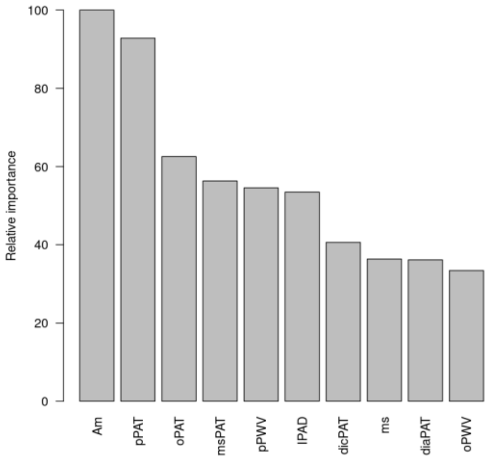

(b)

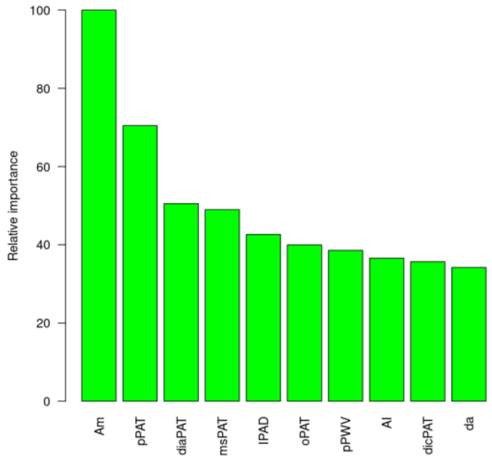

(c)

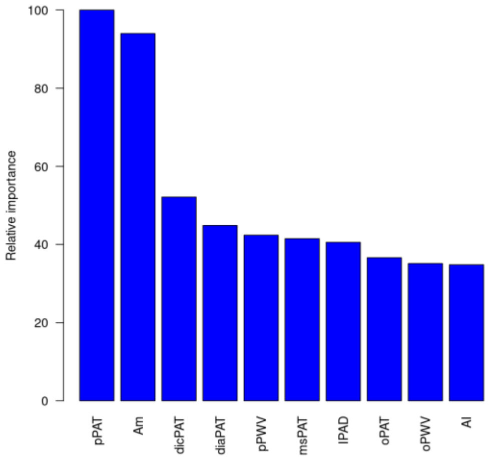

(d)

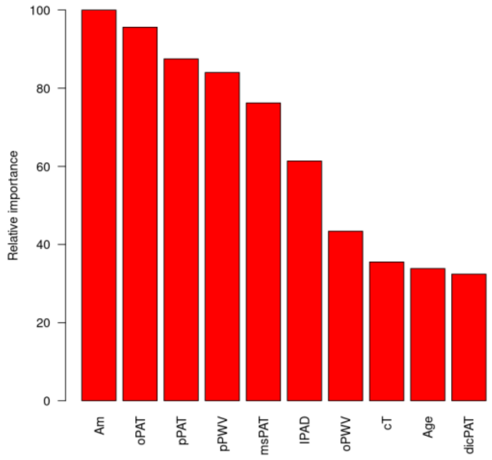

Supplemental digital content 8. DBP importance predictors during WBT. (a) All values; (b) one subject values; (c) Lowest 10% values; (d) Highest 10% values

(a)

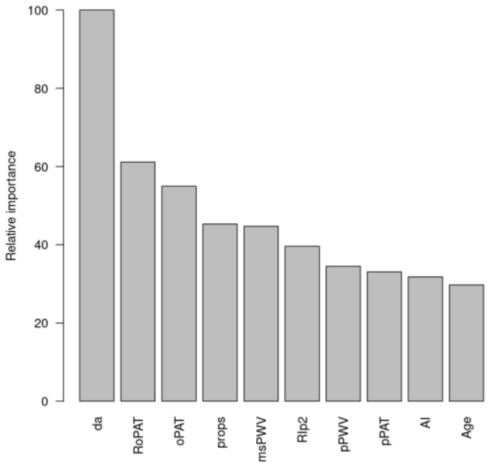

(b)

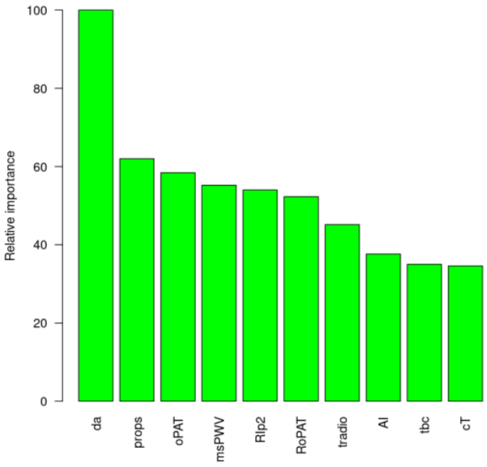

(c)

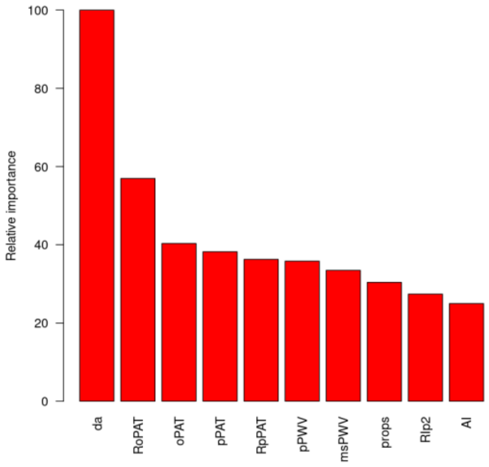

(d)

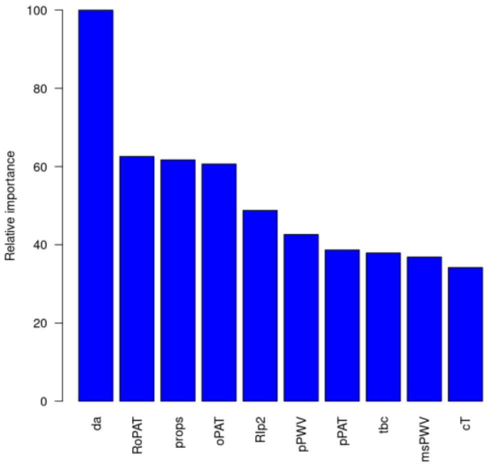

Supplemental digital content 9. SBP importance predictors during WBT. (a) All values; (b) one subject values; (c) Lowest 10% values; (d) Highest 10% values

(a)

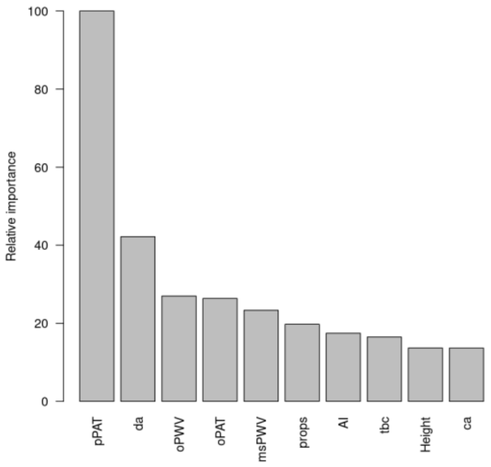

(b)

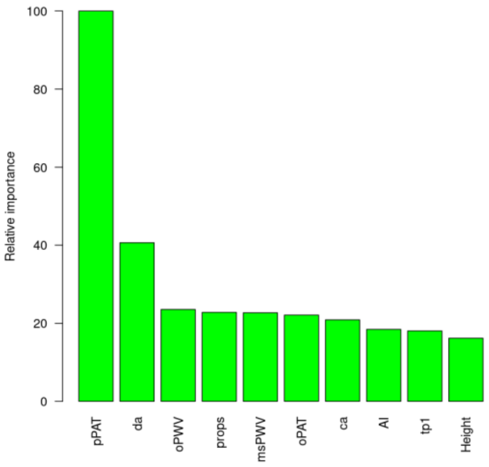

(c)

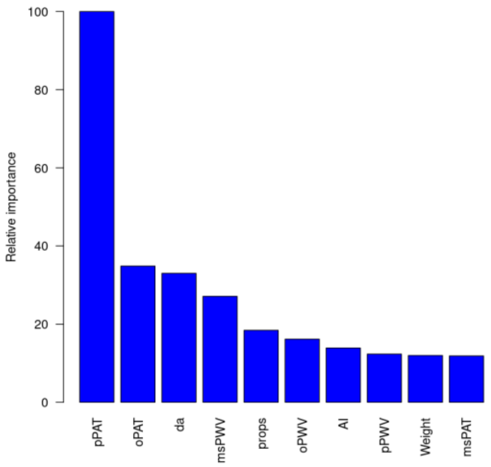

(d)

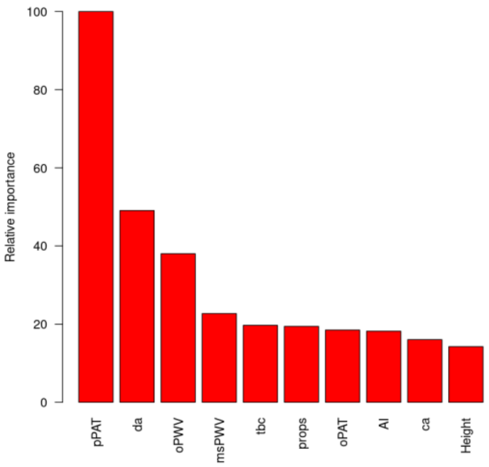

Supplemental digital content 10. MBP importance predictors during WBT. (a) All values; (b) one subject values; (c) Lowest 10% values; (d) Highest 10% values

(a)

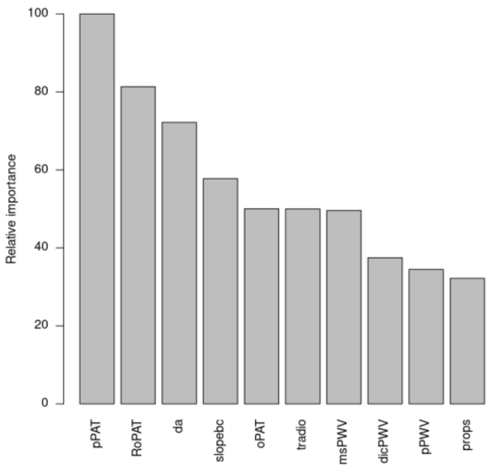

(b)

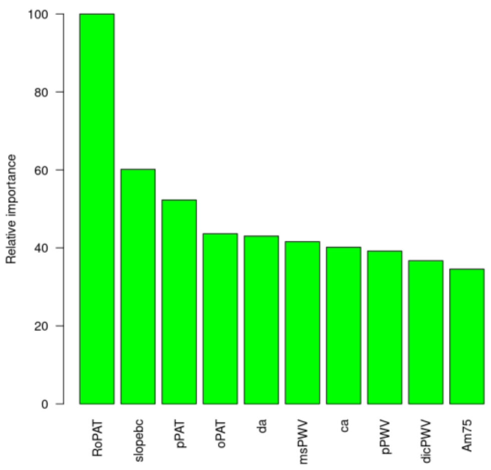

(c)

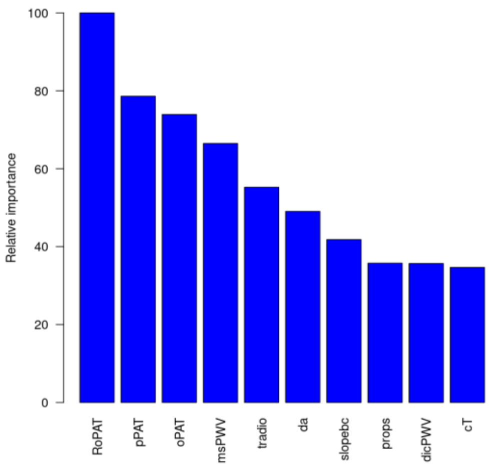

(d)

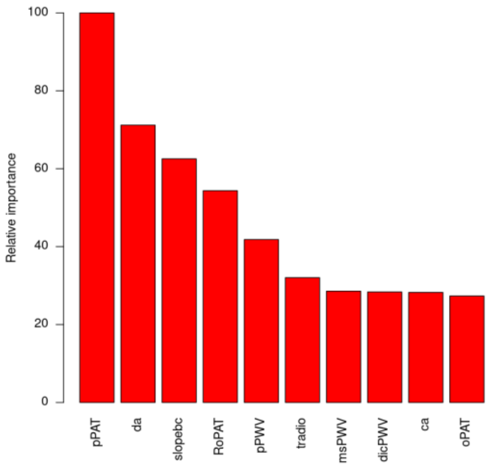

Supplemental digital content 11 Interaction among features for MBP (a) in rest state; (b) during WBT

(a)

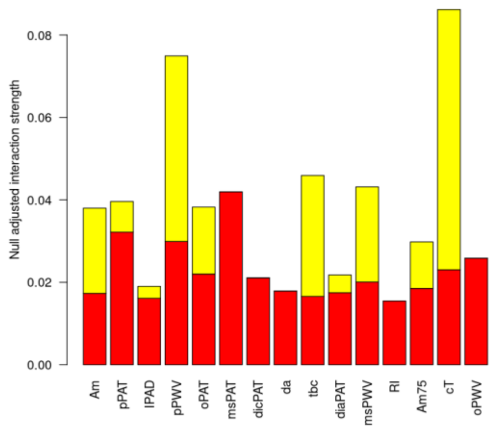

(b)

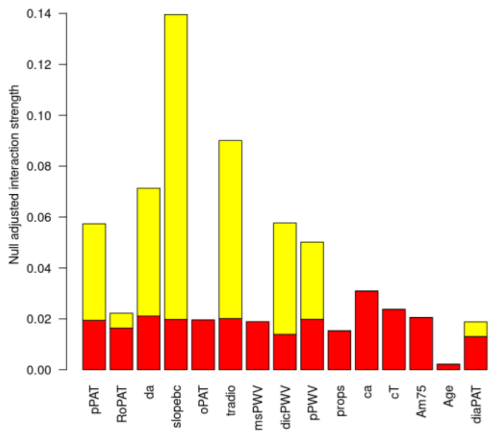

Supplement: Supplementary file 1 [file jcdd-09-00440-s001.zip › jcdd-1907092-supplementary.pdf]
